# Enhancing quinoa growth under severe saline-alkali stress by phosphate solubilizing microorganism *Penicillium funicuiosum* P1

**DOI:** 10.1371/journal.pone.0273459

**Published:** 2022-09-06

**Authors:** Fengyuan Jin, Qilin Hu, Yingxu Zhao, Xiaoyu Lin, Jianfeng Zhang, Jiejing Zhang

**Affiliations:** College of Life Sciences, Key Laboratory of Straw Comprehensive Utilization and Black Soil Conservation, Ministry of Education, Jilin Agricultural University, Changchun, Jilin, China; Savitribai Phule Pune University, INDIA

## Abstract

Promoting the growth of plants and improving plant stress-resistance by plant growth-promoting microorganism increasingly become a hotpot. While, most researchers focus on their supply role of nutrition or plant hormone. In this study, a novel mechanism that phosphate solubilizing microorganisms promoted plant growth under saline-alkali stress through secretion of organic acids, was proposed. The effects of desulfurization gypsum, humic acid, organic fertilizer and phosphate-solubilizing microorganism *Penicillium funicuiosum* P1 (KX400570) on the growth of quinoa (*Chenopodium quinoa* cv. Longli 1), showed that the survival rate, stem length and dry weight of quinoa treated with P1 were 2.5, 1.5, 1 and 1.5 times higher than those of sterile water (CK) under severe saline-alkali stress. The growth-promoting effect of P1 on quinoa was much better than that of other treatment groups. In addition, P1 promoted the growth of quinoa because the organic acids (malic acid, citric acid, succinic acid, etc.) from P1 stimulated the antioxidant system and promote the photosynthesis of quinoa, further promote quinoa growth.

## 1. Introduction

The area of saline-alkali land is about one billion hm^2^ and increases every year [1, 2]. Innutrition [3] and lower diversity or abundance of microorganisms [4] are major characters of saline-alkali soil [5]. Therefore, severe saline-alkali stress endangers plant growth [3] by disturbing photosynthesis [6] and water-uptake [7].

To reduce the stress of saline-alkali soil for plant, the researchers presented lots of methods, including physical [8], chemical [9] and biological methods [10]. Among of them, physical methods, especially salt washing method, worked best [11]. While, its complex operation and high cost hindered their large-scale application [11]. Comparatively, chemical methods were the most common methods for improving saline-alkali soil because of their simple operation and low cost. However, chemical methods such as desulfurization gypsum [12] and humic acid [13], did not work in a short-term [12–14]. The biological methods, including plant of pioneer crops [15], application of microorganism agent [16–18] and organic fertilizer, *etc*. [19–21], worked better to improve saline-alkali soil in a short term. However, biological methods commonly worked on lighter level saline-alkali soil, such as *Chenopodium quinoa*. As a halophyte, *Chenopodium quinoa* grows well and improves soil properties under moderate salt stress (100–200 mM of NaCl) [22]. However, the survival rate is low under high saline-alkali stress. While, many microorganisms with stronger vitality and saline-alkali tolerance can be used to improve higher saline-alkali land. In addition, biological methods are sustainable. Recently, more and more researchers focus on this topic.

Many saline-alkali resistance microorganisms [23] not only reduced soil salinity [24], but also had the functions of nitrogen fixation [25, 26], potassium [27] and phosphate solubilization [10, 28]. Because of their functions, these microorganisms have great application potential on improving the nutrition of saline-alkali soil. Thus, these microorganisms may be used to improve saline-alkali soil [10, 23, 24]. Moreover, many microorganisms secrete bioactive substances such as IAA [28–30], ACC deaminase [31, 32], gibberellin [30], exopolysaccharides [33, 34] and polyamine [35] etc., which activate the antioxidant system of plants and promote plant growth. Therefore, salt-tolerant multifunctional growth-promoting microorganisms have been attracted more attention in recent years [36, 37].

Studies showed that microorganism agent would improve saline-alkali soil properties. Under field experiment, Trichoderma asperellum [38] inoculated into maize decreased the pH of maize rhizosphere soil by 0.25 and increased the contents of organic matter, available nitrogen, phosphorus and potassium in soil as well. Then, the yield of maize increased by 12.41%. Similarly, Jiangbao Xia et.al showed that the microorganisms reduced soil salt content and increased soil available phosphorus and potassium content under filed experiment as well [39]. Further, the microorganisms promoted the uptake of nutrients by Sesbania cannabina and improved the total biomass of Sesbania cannabina by 1.4 times under filed experiment. Besides, microorganisms improved the saturated hydraulic conductivity better than gypsum and helped water transport more smoothly in saline-alkali soil [40].

Microorganism agent not only affect soil properties, but also help plants resist saline-alkali stress by regulating physiological and biochemical reactions in plants. Under saline-alkali stress, *Bacillus licheniformis* activated the pathways related to abiotic stress resistance and iron acquisition in chrysanthemum, and improved the survival rate, photosynthesis and biomass of chrysanthemum [41]. Jiali Liu *et al*. [42] isolated two plant growth promoting strains under saline-alkali stress. After inoculating into alfalfa rhizosphere, the antioxidant system of alfalfa was activated and the activities of antioxidant enzymes were increased. The stem height, shoot dry weight and root dry weight of alfalfa were increased by about 20%, 33% and 100%, respectively. Similarly, Haiyun Li *et al*. [43] inoculated composite microorganism agents into the rhizosphere of oat seedlings. The antioxidant enzymes activities of oat were increased, and the dry weight, plant height and root length of the oat seedlings were increased by about 200%, 100% and 30%, respectively. Interestingly, Chintan Kapadia *et al*. [44] inoculated tomatoes with microbial consortia under salt stress. Compared with the control, the treatment inoculated with microbial consortia did not change soil properties, but increased tomato chlorophyll content and mineral uptake. However, so far, few strains are available for severe saline-alkali soil. P1 has the ability of phosphate solubilization and secreting organic acids in severe saline-alkali soil [45]. Therefore, P1 may benefit for improving saline-alkali soil properties and promoting plant growth. Moreover, helping quinoa adapt to severe saline alkali stress and then improving saline-alkali soil property by microorganisms are still research gap.

Herein, we studied the performance of phosphate-solubilizing fungi P1 to improve severe saline-alkali soil and promote the growth of quinoa. Hence, we illustrated the reason why P1 promoted the growth of quinoa under severe saline-alkali stress through the comparison of desulfurization gypsum, humic acid and organic fertilizer treatments.

## 2. Materials and methods

### 2.1 Materials source

Phosphate-solubilizing fungi Penicillium funicuiosum P1 (KX400570) was preserved in our lab, and the organic acids secreted by P1 were determined qualitatively and quantitatively by liquid chromatograph Ultimate3000 [45]. The organic fertilizer was also prepared ourselves [46]. Quinoa used in this paper was *Chenopodium quinoa* cv. Longli 1, which was bred by Pasture and Green Agriculture Institute of Gansu Academy of Agricultural Sciences. And the quinoa seeds were bought from Shanxi Jinnongyuan Land Products Co., Ltd.. Desulfurized gypsum and humic acid were purchased from Liyuan Environmental Protection Materials Co., Ltd. and Shenzhen Dugao New Biological Technology Co., Ltd., respectively.

### 2.2 Plant growth conditions and treatment

**Pot experiments.** The saline-alkali soil used in pot experiments was collected from the surface 0–20 cm depth of Liujia saline-alkali soil (44°13 ’ 59.559’E, 125°6 ’ 41.349’N) in Nong ’ an County, Changchun City, Jilin Province, China. The soil was dried and separated by a 0. 9 mm sieve, and then put into no-hole pots. The maximum, minimum diameter and height of the pots are 18.5 cm, 10 cm and 11cm, respectively. After that, different treatments were applied to the soil, including sterile water (CK), desulfurized gypsum (FGD), commercial amendments (MA, humic acid content ≥ 70%, 3% of the soil), organic fertilizer (OF, 3% of the soil) and phosphate-solubilizing fungi P1 (P1, 20 mL bacterial solution per 1 kg soil after PDA culture), respectively. Quinoa seeds sterilized were planted in pots (1 kg soil per pot, 35 seeds per pot, 3 replicates). The plants were placed in a greenhouse with a room temperature of 25°C, and the light / dark cycle was 16 h / 8 h. Samples were tested after 30 days. And each test was repeated triple.

**Soilless culture experiment.** Quinoa seeds sterilized were seeded in a 200 mL container with vermiculite. Each container was input suitable Na_2_CO_3_- NaHCO_3_ buffer solution (0.1 M Na_2_CO_3_: 0.1 M Na_2_HCO_3_ = 5: 5) and Hoagland solution. Then five exogenous organic acids were added to the system, which were oxalic acid, acetic acid, succinic acid, citric acid and malic acid. The type and concentration of these organic acids were determined by the secretion of P1. And then 10 quinoa seeds were planted in each container. Each treatment has three parallel samples.

### 2.3 Biochemical analysis

Chlorophyll content of quinoa was determined by portable chlorophyll meter (TYS-4N, Beijing Zhongkeweihe Instrument Company, China). Three mature leaves of three quinoa plants were selected, and the average value of each leaf was recorded three times in vivo.

Proline content was tested by the Acidic Ninhydrin Method [47]. 0.5 g of plant leaves were added into 5 mL 3% sulfosalicylic acid solution, and then heated in boiling water for 10 minutes. After that, 2 mL centrifuged supernatant was mixed with 2 mL acetic acid and 2 mL acidic ninhydrin reagent. Then, it was heated in boiling water bath for 30 min. Then, the solution was cooled, then mixed with 4 mL toluene and stood. Finally, the upper solution was taken and tested at 520 nm wavelength by L5S BMS (INESA Analytical Instrument Co., Ltd), and proline content was got from a standard curve prepared using analytical grade proline and expressed as mg/g FW.

Malondialdehyde (MDA) content was tested by thiobarbituric acid (TBA) method. 0.1 g plant samples were added to 5 mL 5% trichloroacetic acid (TCA) solution, and then ground, homogenized and centrifuged. After that, 2 mL supernatant was taken and mixed with 2 mL 0.67% thiobarbituric acid (TBA). Then, the mixing solution was heated in boiling water bath for 30 min, and following with the measurement of absorbance values at 450 nm, 532 nm and 660 nm by L5S BMS (INESA Analytical Instrument Co., Ltd). MDA content was expressed on a nmol/g FW basis [48].

Antioxidant enzyme (POD, SOD) activity was detected by Zhou W *et al*. [49] method. In the SOD experiments, 0.1 g plant samples were grinded with liquid nitrogen, and then washed with 5 mL phosphate buffer (pH 7.8). After centrifugation, the crude enzyme solution was got. Then, 0.05 g crude enzyme solution reacted with SOD reaction solution, under 4000lx light intensity for 20 min. And then, the absorbance was measured at 560 nm by L5S BMS (INESA Analytical Instrument Co., Ltd). SOD reaction solution was prepared by 0.05 mol/L phosphate buffer (pH 7.8), 130 mmol/L methionine solution, 750 μmol/L nitro-blue tetrazolium solution, 100 μmol/L EDTA-Na_2_ solution and 20 μmol/L riboflavin solution, according to the ratio of 5: 1: 1: 1: 1. In the POD experiment, 0.1 g plant samples were grinded with liquid nitrogen, and then washed with 5 mL phosphate buffer (pH 5.5). After centrifugation, the crude enzyme solution was got. Then, 0.1 mL crude enzyme solution was added to POD reaction solution, and then the absorbance was measured at 470 nm by L5S BMS (INESA Analytical Instrument Co., Ltd). POD reaction solution was prepared with 2.9 mL phosphate buffer (pH 5.5), 1.0 mL 2% hydrogen peroxide and 1.0 mL 0.05 mol/L guaiacol, according to the ratio of 5: 1: 1: 1: 1. Each test was repeated triple.

### 2.4 Soil physical and chemical properties

Soil physical and chemical properties include pH, electrical conductivity, HCO_3_^-^ content, organic matter, available nitrogen, available phosphorus and available potassium. Soil pH and electrical conductivity were measured after mixing the soil with deionized water at a ratio of 1: 5 by pH meter (SevenDirect SD20 HA Kit, METTLER TOLEDO company) and conductivity meter (DDS-11A, Shanghai INESA Scientific Instrument Co, Ltd.), respectively [24].

Soil organic matter were tested by K_2_Cr_2_O_7_-H_2_SO_4_ digestion method [50]. 0.5 g soil sample was mixed with 1 mol/L K_2_Cr_2_O_7_ solution. Then, concentrated sulfuric acid and o-phenanthroline indicator was added into the reaction system. And then, the solution was titrated with 0.5 mol/L FeSO_4_ till brick red. Finally, record the consumption of FeSO_4_ and calculate the content of organic matter through the consumption.

Available nitrogen was tested by alkali-diffusion method [51]. 2.0 g soil sample was evenly mixed with 10 mL 1 mol/L NaOH in the external chamber of the diffusion dish, and then 2 mL boric acid indicator was put into the internal chamber. Then, the reaction was done at 40°C for 24 h. After that, the reaction solution was titrated with 0.01 mol/L H_2_SO_4_ till yellow. Finally, record the consumption of H_2_SO_4_ and calculate the content of organic matter through the consumption.

Available phosphorus was tested by molybdenum antimony colorimetric method [52]. 2.5 g soil samples were added in 0. 5 mol/L NaHCO_3_ to extract available phosphorus, and then the filtrate was reacted with molybdenum antimony reagent for 30 min, and read absorbance at 880 nm by L5S BMS (INESA Analytical Instrument Co., Ltd). Then, available phosphorus concentration was determined according to the standard curve.

Available potassium was tested by flame photometry [53]. Available potassium was extracted from 5.0 g soil sample by neutral NH_4_OAc solution. Then, the soil filtrate and a series of potassium standard solutions were determined by 6400A flame photometer (INESA Analytical Instrument Co., Ltd. And then, standard curve was established, and potassium concentration in filtrate was determined according to standard curve. Each test was repeated triple.

### 2.5 Statistical analysis

One-way ANOVA were determined using SPSS Statistics 25 (IBM, New York, USA). The PCoA was used to express the impact of different treatment methods on soil physical and chemical properties and plants by principal coordinates using a Bray-Curtis dissimilarity matrix calculated from the taxonomic abundance matrices. The PCoA plots were generated using the vegan package [54] in R studio (version 4.1.1).

## 3. Results and discussion

**Fig 1:**
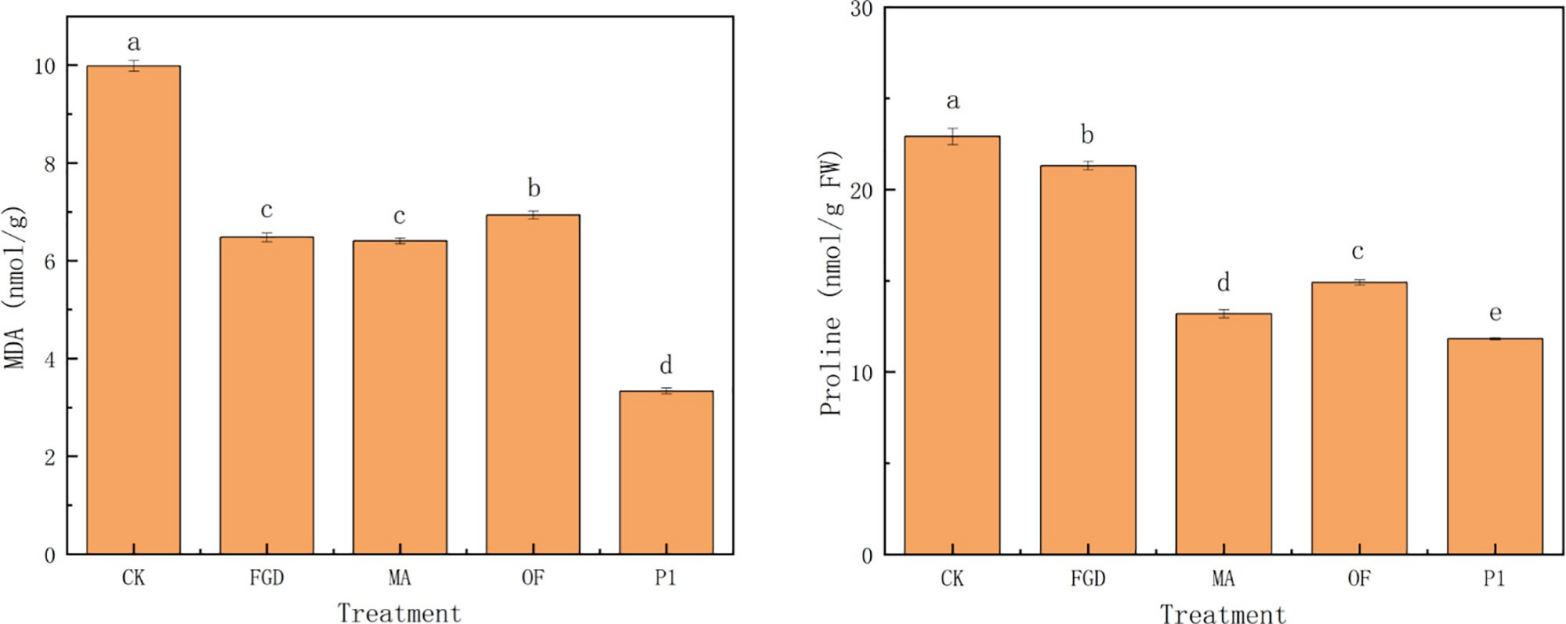
Treatments on MDA and proline of quinoa. CK: sterile water, FGD: desulfurized gypsum, MA: commercial amendments (humic acid content ≥ 70%, 3% of the soil), OF: organic fertilizer (3% of the soil), P1: phosphate-solubilizing fungi P1(20 mL bacterial solution per 1 kg soil after PDA culture). Error bars represent the standard deviation of the replicates (n = 3). Different letters within a column indicate significant differences at P<0.05.

Salinity-alkali stress seriously inhibits the growth of plants [5, 55]. Generally, amendments are applied to improve saline-alkali soil under saline-alkali stress [9, 13, 39, 56]. However, chemical amendments work little on plant growth in a short term under saline-alkali stress [9, 14]. From Table 1, although the MDA content (Fig 1A) and proline content (Fig 1B) were decreased after the application of desulfurization gypsum and humic acid, the growth of quinoa seedlings were not significantly improved. The results have the same tendency with that of Yonggan Zha *et al*. [12] and Hu, Y. W. et al. [13]. While, organic fertilizer works better on quinoa’s growth. After organic fertilizer treatment, quinoa survival rate, root and stem length, fresh and dry weight, were increased by about 2 times, 60% and 1 times compared with CK, respectively (Table 1). Moreover, the content of both MDA (Fig 1A) and proline (Fig 1B) decreased by about 65%. This indicated that organic fertilizer treatment not only alleviated saline-alkali stress, but also effectively promoted quinoa growth. Why did organic fertilizer improve quinoa growth?

**Table 1:** Growth indicators of quinoa under different treatments.

Treatment	Survival rate (%)	Fresh weight(mg/plant)	Dry weight(mg/plant)	Root length(cm)	Stem length(cm)
**CK**	10.48±1.65b	90.7	7.6	4.02±0.38d	4.99±0.99b
**FGD**	16.19±1.65b	97.7	8.6	4.64±0.39c	5.65±0.72b
**MA**	13.33±3.30b	147.5	13.2	4.66±0.38c	5.53±0.67b
**OF**	29.52±4.36a	171.7	17.1	7.44±0.57b	7.96±0.65a
**P1**	34.29±5.71a	187.4	19.1	10.12±0.45a	7.76±0.68a

CK: sterile water, FGD: desulfurized gypsum, MA: commercial amendments (humic acid content ≥ 70%, 3% of the soil), OF: organic fertilizer (3% of the soil), P1: phosphate-solubilizing fungi P1(20 mL bacterial solution per 1 kg soil after PDA culture). Means ± standard deviations for three replicates. Different letters within a column indicate significant differences at P<0.05.

Recent studies provided possible answers: organic fertilizers were beneficial for optimizing saline-alkali soil microbial community [20, 38]. Organic fertilizer provided nutrients for microorganisms and then increased the abundance of beneficial microorganisms [20, 57]. And then, thanks to these beneficial microorganisms, organic fertilizer plays a role on assisting plants to resist saline-alkali stress [19, 58]. In another words, beneficial microorganisms were able to help plants resist saline-alkali stress [34]. Then, will it be better if we apply beneficial microorganisms directly to the saline-alkali soil?

To solve the problem, we studied the effect of phosphate-solubilizing microorganism P1 on assisting quinoa to resist saline-alkali stress and its mechanism. As shown in Table 1, the treatment inoculated with P1 better promote the growth of quinoa under severe saline-alkali stress than organic fertilizer. The survival rate of quinoa increased by about 20% (Table 1). Both fresh weight and dry weight of quinoa increased by about 10% (Table 1). Furthermore, compared with organic fertilizer, MDA (Fig 1A) and proline (Fig 1B) content of quinoa decreased by 52% and 21%, respectively. Thus, beneficial microorganisms were more favorable for promoting plant growth.

In summary, soil amendments were beneficial for promoting the growth of quinoa under saline-alkali stress. Among them, microbial agent P1 worked best. P1 increased the survival rate, root and stem length, fresh and dry weight per plant by 2.5 times, 1.5 times, 55%, 1 time and 1.5 times, respectively, under severe saline-alkali stress. The experimental results were consistent with those of Lei Kong *et al*. [59] and Mehrnoush Eskandari Torbaghan *et al*. [60]. Lei Kong *et al*. [59] inoculated AMF fungi into saline-alkali soil. And the stem length of tomato was increased by 39.32%. But, P1 increased the stem length of quinoa by 55%. Mehrnoush Eskandari Torbagha *et al*. [60] inoculated *Alkalibacillus haloalkaliphilus* in saline-alkali soil. And the fresh weight of wheat was increased by about 70%. But, P1 doubled the fresh weight of quinoa. What caused the remarkable effect of phosphate-solubilizing microorganisms P1 on quinoa?

**Fig 2:**
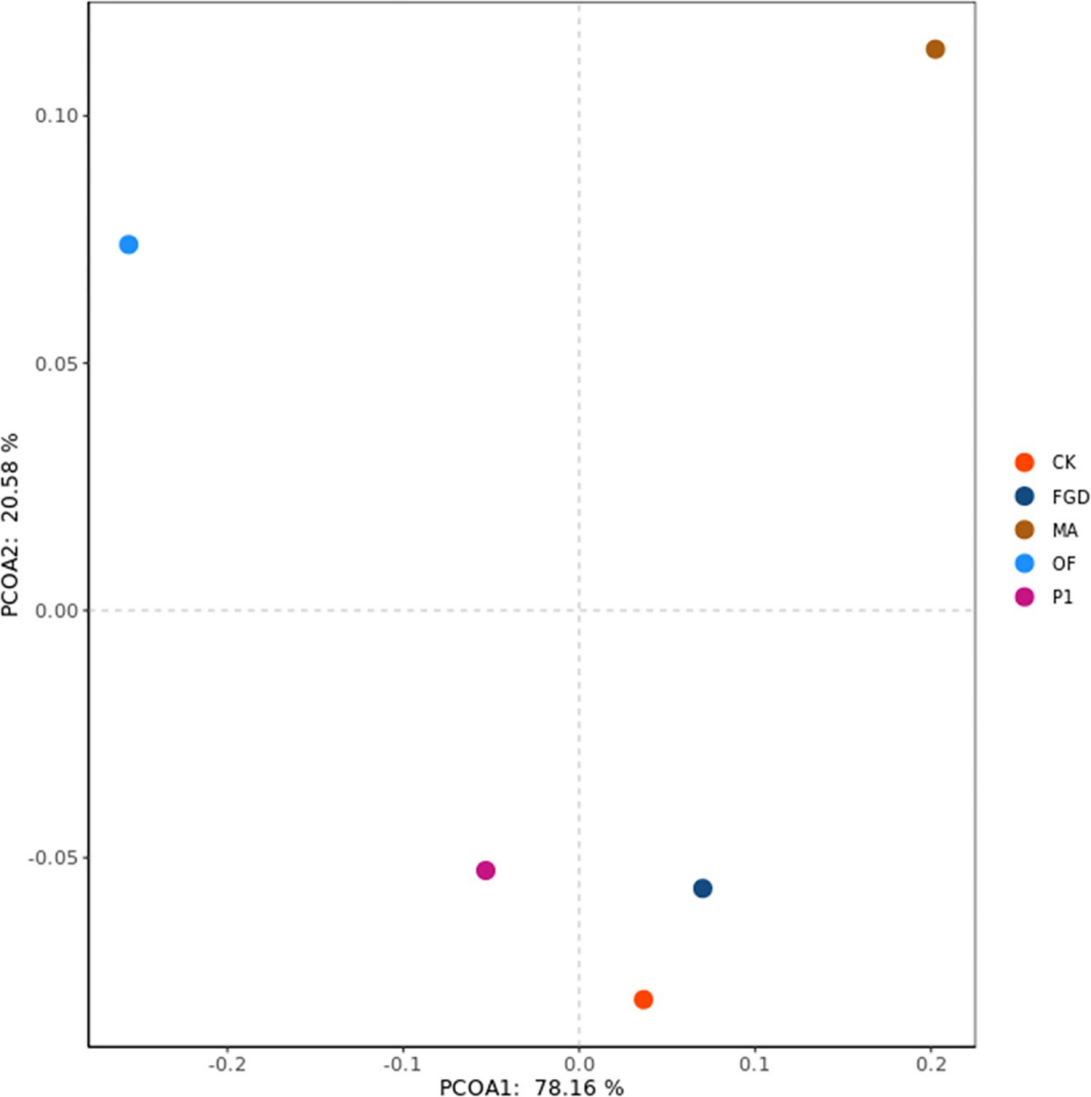
Difference visualization of saline-alkali soil physical and chemical properties under different treatments. CK: sterile water, FGD: desulfurized gypsum, MA: commercial amendments (humic acid content ≥ 70%, 3% of the soil), OF: organic fertilizer (3% of the soil), P1: phosphate-solubilizing fungi P1(20 mL bacterial solution per 1 kg soil after PDA culture). The data comes from all the data about the physical and chemical properties of soil mentioned in this paper. Principal coordinates use a Bray-Curtis dissimilarity matrix calculated from the taxonomic abundance matrices.

**Fig 3:**
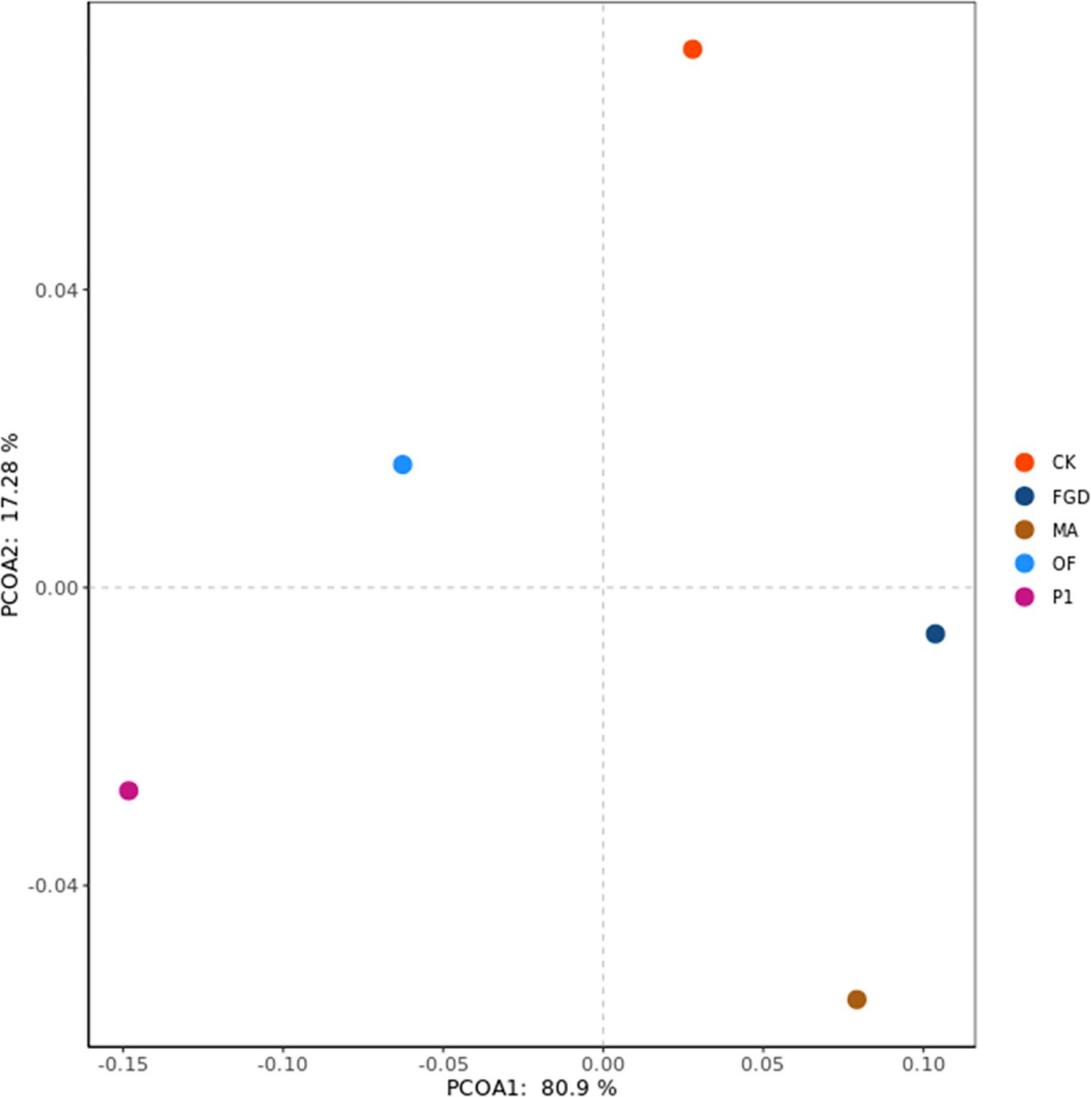
Difference visualization of quinoa growth under different treatments. CK: sterile water, FGD: desulfurized gypsum, MA: commercial amendments (humic acid content ≥ 70%, 3% of the soil), OF: organic fertilizer (3% of the soil), P1: phosphate-solubilizing fungi P1(20 mL bacterial solution per 1 kg soil after PDA culture). The data comes from all the data about the quinoa mentioned in this paper. Principal coordinates use a Bray-Curtis dissimilarity matrix calculated from the taxonomic abundance matrices.

Previous researches illustrated that phosphate-solubilizing microorganisms can improve soil properties and provide suitable growth environment for plant growth [61–63]. Fungi P1 had similar function as well. P1 secreted organic acids during their growth process. These organic acids decreased soil pH and bicarbonate concentration (Table 2). But improvement of soil properties was impossible for the reason of quinoa growth. From the statistical analysis, organic fertilizer treatment performed best on improving saline-alkali soil properties, followed by humic acid, as shown in Fig 2. However, the treatment with P1 works the best on the growth of quinoa (Fig 3). This phenomenon indicated that some important factors were ignored. Then, whether organic acids secreted by P1 not only reduced the saline-alkali soil pH, but also promoted plant growth?

**Table 2:** Physical and chemical properties of saline-alkali soil under different treatments.

Treatment	pH	EC (mS/cm)	HCO_3_^-^(mg/kg)	Available-N (mg/kg)	Available-P (mg/kg)	Available-K (mg/kg)	Organic carbon (g/kg)
**CK**	9.12±0.02ab	0.96±0.02d	48.80±3.05a	130.67±5.35b	14.39±0.30e	268.92±27.56b	7.85±0.83b
**FGD**	9.15±0.02a	1.02±0.01cd	47.78±1.76a	120.17±5.35b	16.98±0.36c	179.28±11.02c	5.59±0.08c
**MA**	8.51±0.02d	1.23±0.01b	28.47±1.76c	75.83±5.35c	27.63±0.28b	268.9±25.51b	5.72±0.61c
**OF**	9.06±0.04b	1.55±0.03a	41.68±1.76b	175.00±9.26a	151.32±0.36a	494.97±16.54a	20.48±1.51a
**P1**	8.77±0.08c	1.08±0.0c	40.67±1.76b	166.83±2.02a	15.71±0.36d	183.18±5.51c	9.71±0.83b

CK: sterile water, FGD: desulfurized gypsum, MA: commercial amendments (humic acid content ≥ 70%, 3% of the soil), OF: organic fertilizer (3% of the soil), P1: phosphate-solubilizing fungi P1(20 mL bacterial solution per 1 kg soil after PDA culture). Means ± standard deviations for three replicates. Different letters within a column indicate significant differences at P<0.05.

**Fig 4:**
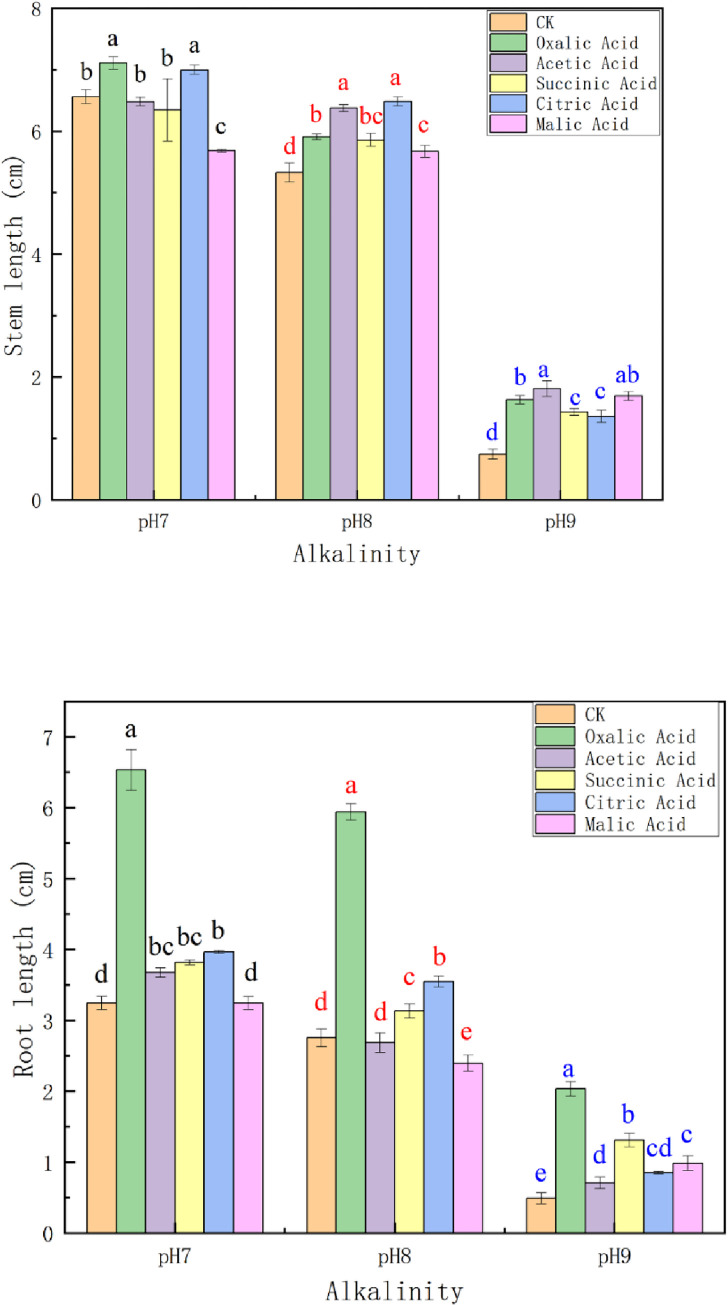
Effects of different organic acids on stem length (a) and root length (b) of quinoa. CK: Hoagland solution, Oxalic Acid: Hoagland solution and oxalic acid, Acetic Acid: Hoagland solution and acetic acid, Succinic Acid: Hoagland solution and succinic acid, Citric Acid: Hoagland solition and citric acid, Malic acid: Hoagland solution and malic acid. Error bars represent the standard deviation of the replicates (n = 3). Different letters within a column indicate significant differences at P<0.05.The color of letters represents the significant difference that they belong to the same group.

Some exogenous organic acids promoted the growth of plants. Some exogenous organic acids such as succinic acid increased organic acids (succinic acid, citric acid, malic acid and oxalic acid, *etc*.) in plant cytoplasm [64]. The increase of intracellular organic acid content was beneficial for plants to resist saline-alkali stress especially oxalic acid [65], and then resulted in promoting plant growth [66, 67]. Does P1 secret similar organic acids? In order to solve this problem, we detected the organic acids secreted by P1. As shown in Table 3, P1 secretes a variety of organic acids, among which oxalic acid, acetic acid, citric acid, malic acid and succinic acid are abundant. Therefore, we studied the effects of exogenous oxalic acid, acetic acid, citric acid, malic acid and succinic acid on the growth of quinoa, respectively (Fig 4). The results showed that all these acids had obvious growth-promoting effects on quinoa under different pH buffer conditions. Moreover, the growth-promoting effect of organic acids on plants increased with the increase of pH. Among them, oxalic acid, acetic acid and malic acid had better growth-promoting effect on quinoa. In pH 9 buffer solution, the stem length and root length of quinoa in oxalic acid treatment group increased by 1.5 and 3 times, respectively. Both stem and root length of quinoa in the acetic acid and malic acid treatment groups both increased by about 1.5 times.

**Table 3:** Organic acids produced by P1.

Organic acid	Concentration (mg/L)	Molarity (mM/L)
Oxalic Acid	763	8.4741
Acetic Acid	154.6795	2.5758
Succinic Acid	209.1741	1.7713
Citric Acid	247.1276	1.2863
Lactic acid	69.0017	0.7660
Formic acid	12.4190	0.2698
Propionic acid	19.8204	0.2676
Malic acid	20.0531	0.1495
VC	6.4761	0.0367689
Tartaric acid	5.4689	0.0364375

Qualitative and quantitative analysis of organic acids were used by high performance liquid chromatography.

**Fig 5:**
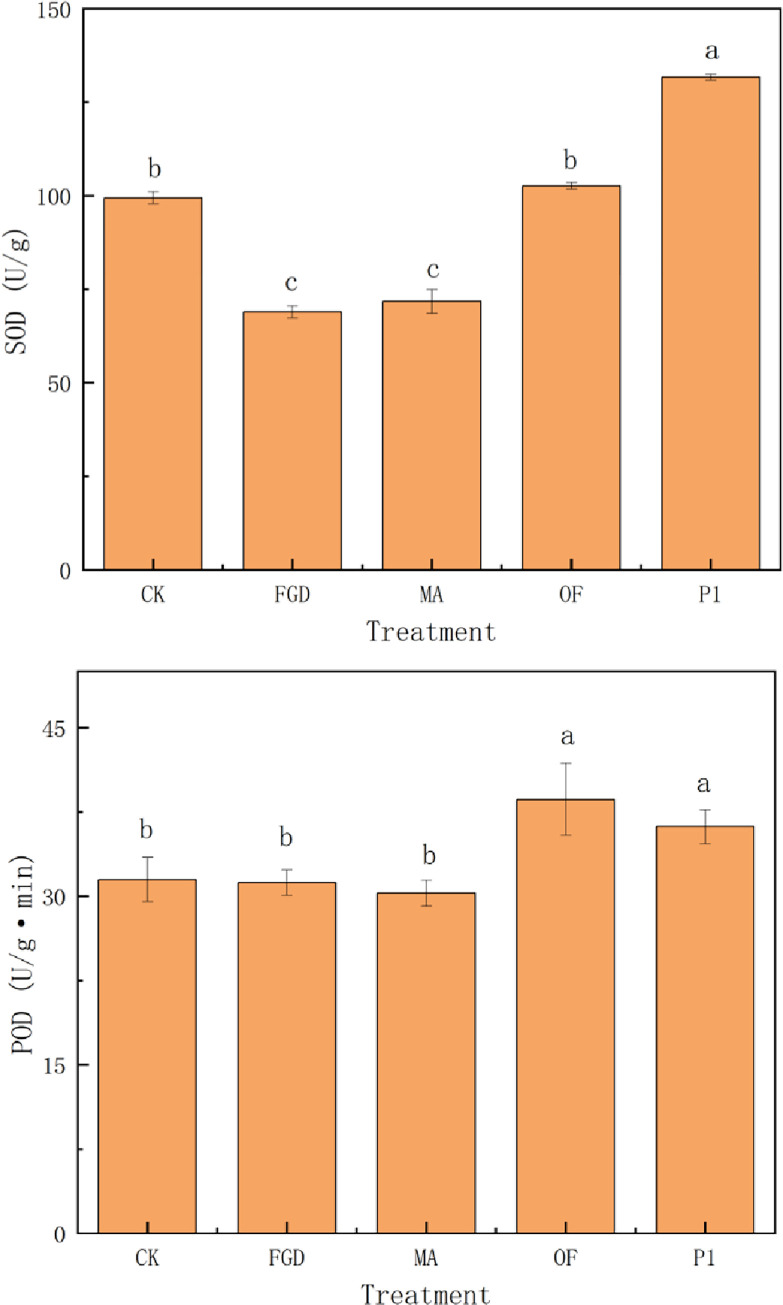
Effects of different treatments on antioxidant enzymes in quinoa. CK: sterile water, FGD: desulfurized gypsum, MA: commercial amendments (humic acid content ≥ 70%, 3% of the soil), OF: organic fertilizer (3% of the soil), P1: phosphate-solubilizing fungi P1(20 mL bacterial solution per 1 kg soil after PDA culture). Error bars represent the standard deviation of the replicates (n = 3). Different letters within a column indicate significant differences at P<0.05.

**Fig 6:**
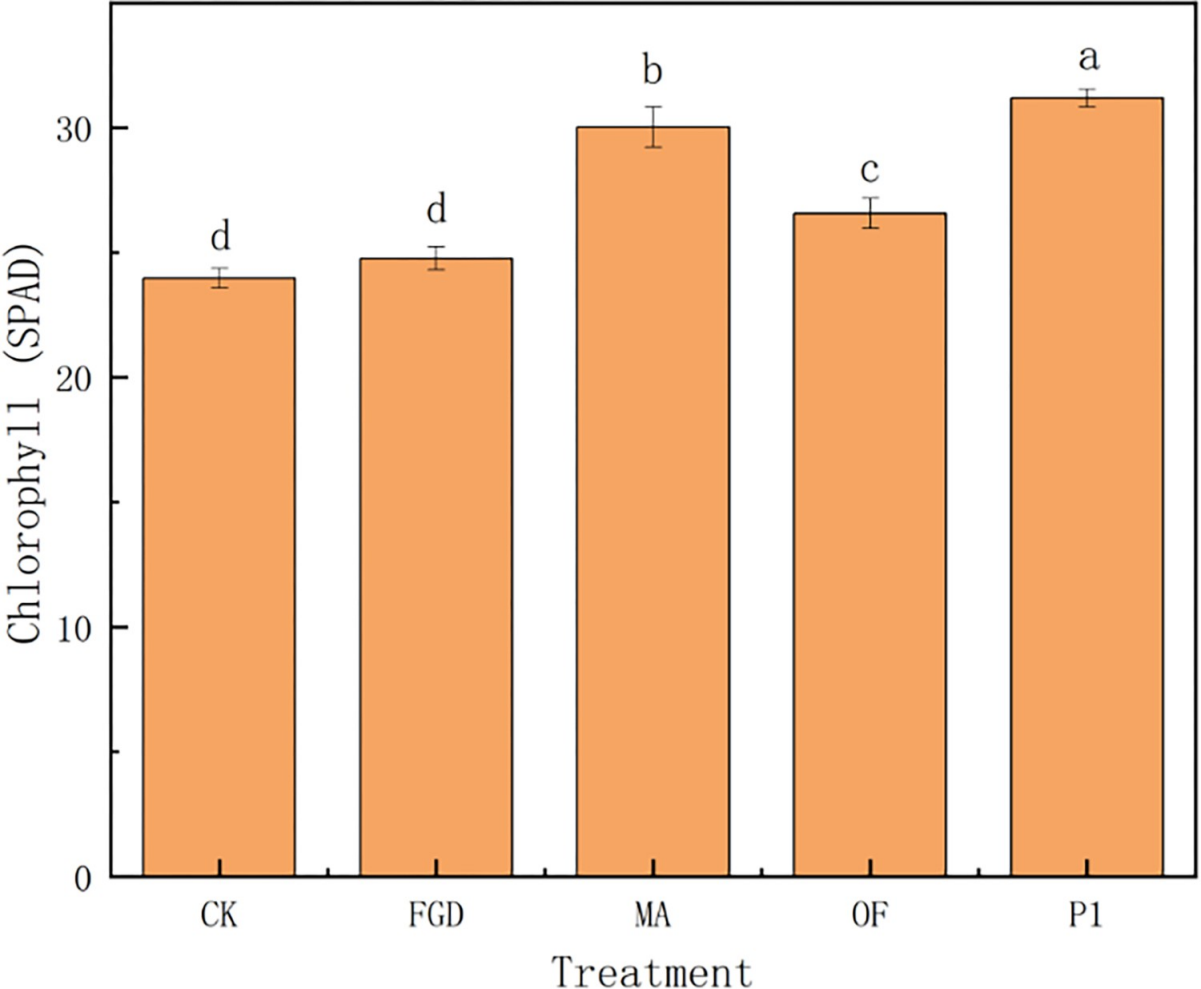
Effects of different treatments on chlorophyll content in quinoa. CK: sterile water, FGD: desulfurized gypsum, MA: commercial amendments (humic acid content ≥ 70%, 3% of the soil), OF: organic fertilizer (3% of the soil), P1: phosphate-solubilizing fungi P1(20 mL bacterial solution per 1 kg soil after PDA culture). Error bars represent the standard deviation of the replicates (n = 3). Different letters within a column indicate significant differences at P<0.05.

Organic acids promoted plant growth may due to the following two reasons. Firstly, organic acids activated the antioxidant system of plants under stress, and improved the tolerance of plants to abiotic stress. Thus, plant growth was improved. Succinic acid induced the accumulation of succinic acid, citric acid, malic acid and oxalic acid in plants, up-regulated the gene expression of malate dehydrogenase (MDH) and phosphoenolpyruvate carboxylase (PEPC) in plant roots, and effectively alleviated aluminum stress and promote the growth of plants [64]. Oxalic acid and citric acid improved the SOD and POD activities of *Larix olgensis* and activated the antioxidant system of the plants under lead stress and improved the growth of *Larix olgensis* [68]. Citric acid and acetic acid improved the SOD and POD activities of *Melilotus officinalis* under copper stress, and improved the growth of *Melilotus officinalis* [69]. From here we see that the activation of antioxidant system can effectively assist plants to resist stress and promote plant growth. Similar results were obtained in this experiment. The organic acids secreted by P1 also actived the antioxidant system, and increased SOD (Fig 5A) and POD (Fig 5B) in quinoa by 30% and 16%, respectively. Secondly, organic acids promoted photosynthesis of plants. Malic acid enhanced photosynthesis by increasing chlorophyll content [70] or inducing stomatal opening [71]. Acetic acid also enhanced photosynthesis by increasing chlorophyll content [72]. Citric acid enhanced photosynthesis by increasing carotenoid content and the number and volume of chloroplasts in mesophyll cells [70]. Similar phenomenon was observed in our experiment. The chlorophyll content in quinoa treated with P1 was significantly higher than other treatments. (Fig 6) Therefore, organic acids promoting photosynthesis of quinoa is another reason why these organic acids promote quinoa growth. It can be found that the organic acids secreted by P1 played an important role on assisting quinoa to resist saline-alkali stress and promoting its growth.

In summary, the main reason why phosphate-solubilizing microorganisms promote plant growth is that they secrete organic acids. On the one hand, these organic acids reduced soil pH and provided a better growth environment for plants. On the other hand, they affect the metabolic system of plants, improve the resistance capability of plants and promote photosynthesis.

## 4. Conclusion

Different methods of alleviating saline alkali stress have great differences in promoting plant growth, due to different mechanisms. Although the improvement effect on soil was obvious after the application of chemical amendments, the growth of quinoa was only improved little. However, biological methods not only worked on improving saline-alkali soil properties, but also promoted quinoa growth. Both organic fertilizer and P1 microbial agent activated the antioxidant system of quinoa. And the organic acids secreted by P1 not only played a role on soil properties improvement, but also were used as bioactive substances to promote quinoa. We believe that small molecular organic acids which have biological activity secreted by microorganisms play a very important role on plant resistance to stress. In the future, P1 agent is going to applied in open field experiments and the effect of P1 on other plants, such as rice, wheat, corn and so on, will be studied as well. Furthermore, the effect of P1 on soil microorganism community and saline-alkali stress response gene express of plant are going to study.

## Supporting information

S1 Appendix (XLSX)

## References

[1] Montanarella L, Chude V, Yagi K, Krasilnikov P, Others GBAAA. Status of the World’s Soil Resources (SWSR)—Main Report: Status of the World’s Soil Resources (SWSR)—Main Report; 2015.

[2] Kang Y, Khan S, Ma X. Climate change impacts on crop yield, crop water productivity and food security–A review. Progress in Natural Science. 2009;19(12):1665–74. doi: 10.1016/j.pnsc.2009.08.001

[3] Khan N, Ali S, Shahid MA, Mustafa A, Sayyed RZ, Cura JA. Insights into the Interactions among Roots, Rhizosphere, and Rhizobacteria for Improving Plant Growth and Tolerance to Abiotic Stresses: A Review. Cells. 2021;10(6). Epub 2021/07/03. doi: 10.3390/cells10061551 34205352 PMC8234610

[4] Fazeli-Nasab B, Sayyed RZ, Mojahed LS, Rahmani AF, Ghafari M, Antonius S, et al. Biofilm production: A strategic mechanism for survival of microbes under stress conditions. Biocatalysis and Agricultural Biotechnology. 2022;42. doi: 10.1016/j.bcab.2022.102337

[5] Fang S, Hou X, Liang X. Response Mechanisms of Plants Under Saline-Alkali Stress. Front Plant Sci. 2021;12:667458. Epub 2021/06/22. doi: 10.3389/fpls.2021.667458 34149764 PMC8213028

[6] Kaiwen G, Zisong X, Yuze H, Qi S, Yue W, Yanhui C, et al. Effects of salt concentration, pH, and their interaction on plant growth, nutrient uptake, and photochemistry of alfalfa (Medicago sativa) leaves. Plant Signal Behav. 2020;15(12):1832373. Epub 2020/10/20. doi: 10.1080/15592324.2020.1832373 33073686 PMC7671061

[7] Mcgeorge WT. Diagnosis and Improvement of Saline and Alkaline Soils. Soil Science Society of America Journal. 1954;18(3):348.

[8] Wang Z, Heng T, Li W, Zhang J, Zhangzhong L. Effects of subsurface pipe drainage on soil salinity in saline‐sodic soil under mulched drip irrigation. Irrigation and Drainage. 2019;69(1):95–106. doi: 10.1002/ird.2383

[9] Temiz C, Cayci G. The effects of gypsum and mulch applications on reclamation parameters and physical properties of an alkali soil. Environ Monit Assess. 2018;190(6):347. doi: 10.1007/s10661-018-6669-4 29770890

[10] Wang W, Wu Z, He Y, Huang Y, Li X, Ye BC. Plant growth promotion and alleviation of salinity stress in Capsicum annuum L. by Bacillus isolated from saline soil in Xinjiang. Ecotoxicol Environ Saf. 2018;164:520–9. Epub 2018/08/28. doi: 10.1016/j.ecoenv.2018.08.070 . 30149350

[11] Christen E, Skehan D. Design and Management of Subsurface Horizontal Drainage to Reduce Salt Loads. Journal of Irrigation & Drainage Engineering. 2001;127(3):148–55.

[12] Zhao Y, Wang S, Li Y, Liu J, Zhuo Y, Zhang W, et al. Long-term performance of flue gas desulfurization gypsum in a large-scale application in a saline-alkali wasteland in northwest China. Agriculture, Ecosystems & Environment. 2018;261:115–24. doi: 10.1016/j.agee.2018.01.009

[13] Hu YW, Li QK, Song CJ, Jin XH. Effect of Humic Acid Combined with Fertilizer on the Improvement of Saline-Alkali Land and Cotton Growth. Applied Ecology and Environmental Research. 2021;19(2):1279–94. doi: 10.15666/aeer/1902_12791294

[14] Wang SJ, Chen Q, Li Y, Zhuo YQ, Xu LZ. Research on saline-alkali soil amelioration with FGD gypsum. Resources, Conservation and Recycling. 2017;121:82–92. doi: 10.1016/j.resconrec.2016.04.005

[15] Ventura Y, Eshel A, Pasternak D, Sagi M. The development of halophyte-based agriculture: past and present. Ann Bot. 2015;115(3):529–40. Epub 2014/08/15. doi: 10.1093/aob/mcu173 25122652 PMC4332600

[16] Cubillos Hinojosa JG, Valero Valero NO, Peralta ADJ. Effect of a low rank coal inoculated with coal solubilizing bacteria for the rehabilitation of a saline-sodic soil in field conditions. Revista Facultad Nacional de Agronomía. 2017;70(3):8271–83. doi: 10.15446/rfna.v70n3.62478

[17] Basu A, Prasad P, Das SN, Kalam S, Sayyed RZ, Reddy MS, et al. Plant Growth Promoting Rhizobacteria (PGPR) as Green Bioinoculants: Recent Developments, Constraints, and Prospects. Sustainability. 2021;13(3). doi: 10.3390/su13031140

[18] Hamid B, Zaman M, Farooq S, Fatima S, Sayyed RZ, Baba ZA, et al. Bacterial Plant Biostimulants: A Sustainable Way towards Improving Growth, Productivity, and Health of Crops. Sustainability. 2021;13(5). doi: 10.3390/su13052856

[19] Shi S, Tian L, Nasir F, Bahadur A, Batool A, Luo S, et al. Response of microbial communities and enzyme activities to amendments in saline-alkaline soils. Applied Soil Ecology. 2019;135:16–24. doi: 10.1016/j.apsoil.2018.11.003

[20] Wu Y, Li Y, Zheng C, Zhang Y, Sun Z. Organic amendment application influence soil organism abundance in saline alkali soil. European Journal of Soil Biology. 2013;54:32–40. doi: 10.1016/j.ejsobi.2012.10.006

[21] Fallah M, Hadi H, Amirnia R, Hassanzadeh-Ghorttapeh A, Zuan ATK, Sayyed RZ. Eco-friendly soil amendments improve growth, antioxidant activities, and root colonization in lingrain (Linum Usitatissimum L.) under drought conditions. PLoS One. 2021;16(12):e0261225. doi: 10.1371/journal.pone.0261225 34941919 PMC8700020

[22] Cai D, Xu Y, Zhao F, Zhang Y, Duan H, Guo X. Improved salt tolerance of Chenopodium quinoa Willd. contributed by Pseudomonas sp. strain M30-35. PeerJ. 2021;9:e10702. doi: 10.7717/peerj.10702 33520465 PMC7811290

[23] Liu HQ, Lu XB, Li ZH, Tian CY, Song J. The role of root‐associated microbes in growth stimulation of plants under saline conditions. Land Degradation & Development. 2021;32(13):3471–86. doi: 10.1002/ldr.3955

[24] Hinsinger P, Plassard C, Tang C, Jaillard B. Origins of root-mediated pH changes in the rhizosphere and their responses to environmental constraints: A review. Plant and Soil. 2003;248(1):43–59. doi: 10.1023/A:1022371130939

[25] Song T, Xu H, Sun N, Jiang L, Tian P, Yong Y, et al. Metabolomic Analysis of Alfalfa (Medicago sativa L.) Root-Symbiotic Rhizobia Responses under Alkali Stress. Front Plant Sci. 2017;8:1208. Epub 2017/07/27. doi: 10.3389/fpls.2017.01208 28744296 PMC5504246

[26] Bertrand A, Gatzke C, Bipfubusa M, Lévesque V, Chalifour FP, Claessens A, et al. Physiological and Biochemical Responses to Salt Stress of Alfalfa Populations Selected for Salinity Tolerance and Grown in Symbiosis with Salt-Tolerant Rhizobium. Agronomy. 2020;10(4). doi: 10.3390/agronomy10040569

[27] Baba ZA, Hamid B, Sheikh TA, Alotaibi SH, El Enshasy HA, Ansari MJ, et al. Psychrotolerant Mesorhizobium sp. Isolated from Temperate and Cold Desert Regions Solubilizes Potassium and Produces Multiple Plant Growth Promoting Metabolites. Molecules. 2021;26(19). doi: 10.3390/molecules26195758 34641302 PMC8510370

[28] Jabborova D, Annapurna K, Fayzullaeva M, Sulaymonov K, Kadirova D, Jabbarov Z, et al. Isolation and characterization of endophytic bacteria from ginger (Zingiber officinale Rosc.). Annals of Phytomedicine: An International Journal. 2020;9(1). doi: 10.21276/ap.2020.9.1.14

[29] Li M, Wang J, Yao T, Wang Z, Zhang H, Li C. Isolation and Characterization of Cold-Adapted PGPB and Their Effect on Plant Growth Promotion. J Microbiol Biotechnol. 2021;31(9):1218–30. doi: 10.4014/jmb.2105.05012 34261854 PMC9705895

[30] Akhtar N, Ilyas N, Yasmin H, Sayyed RZ, Hasnain Z, E AE, et al. Role of Bacillus cereus in Improving the Growth and Phytoextractability of Brassica nigra (L.) K. Koch in Chromium Contaminated Soil. Molecules. 2021;26(6). doi: 10.3390/molecules26061569 33809305 PMC7998664

[31] Sagar A, Sayyed RZ, Ramteke PW, Sharma S, Marraiki N, Elgorban AM, et al. ACC deaminase and antioxidant enzymes producing halophilic Enterobacter sp. PR14 promotes the growth of rice and millets under salinity stress. Physiol Mol Biol Plants. 2020;26(9):1847–54. doi: 10.1007/s12298-020-00852-9 32943820 PMC7468042

[32] Sagar A, Riyazuddin R, Shukla P, Ramteke P, Sayyed R. Heavy metal stress tolerance in Enterobacter sp. PR14 is mediated by plasmid. Indian journal of experimental biology. 2020:115–21.

[33] Ilyas N, Mumtaz K, Akhtar N, Yasmin H, Sayyed RZ, Khan W, et al. Exopolysaccharides Producing Bacteria for the Amelioration of Drought Stress in Wheat. Sustainability. 2020;12(21). doi: 10.3390/su12218876

[34] Kalam S, Basu A, Ahmad I, Sayyed RZ, El-Enshasy HA, Dailin DJ, et al. Recent Understanding of Soil Acidobacteria and Their Ecological Significance: A Critical Review. Front Microbiol. 2020;11:580024. doi: 10.3389/fmicb.2020.580024 33193209 PMC7661733

[35] Zhou C, Ma Z, Zhu L, Xiao X, Xie Y, Zhu J, et al. Rhizobacterial Strain Bacillus megaterium BOFC15 Induces Cellular Polyamine Changes that Improve Plant Growth and Drought Resistance. Int J Mol Sci. 2016;17(6). doi: 10.3390/ijms17060976 27338359 PMC4926508

[36] Sagar A, Rai S, Ilyas N, Sayyed RZ, Al-Turki AI, El Enshasy HA, et al. Halotolerant Rhizobacteria for Salinity-Stress Mitigation: Diversity, Mechanisms and Molecular Approaches. Sustainability. 2022;14(1). doi: 10.3390/su14010490

[37] Sagar A, Yadav SS, Sayyed RZ, Sharma S, Ramteke PW. Bacillus subtilis: A Multifarious Plant Growth Promoter, Biocontrol Agent, and Bioalleviator of Abiotic Stress. In: Islam MT, Rahman M, Pandey P, editors. Bacilli in Agrobiotechnology: Plant Stress Tolerance, Bioremediation, and Bioprospecting. Cham: Springer International Publishing; 2022. p. 561–80.

[38] Fu J, Xiao Y, Liu Z, Zhang Y, Wang Y, Yang K. Trichoderma asperellum improves soil microenvironment in different growth stages and yield of maize in saline-alkaline soil of the Songnen Plain. Plant, Soil and Environment. 2020;66(No. 12):639–47. doi: 10.17221/456/2020-pse

[39] Cui Q, Xia J, Yang H, Liu J, Shao P. Biochar and effective microorganisms promote Sesbania cannabina growth and soil quality in the coastal saline-alkali soil of the Yellow River Delta, China. Sci Total Environ. 2021;756:143801. doi: 10.1016/j.scitotenv.2020.143801 33307496

[40] Sahin U, Eroğlu S, Sahin F. Microbial application with gypsum increases the saturated hydraulic conductivity of saline–sodic soils. Applied Soil Ecology. 2011;48(2):247–50. doi: 10.1016/j.apsoil.2011.04.001

[41] Zhou C, Zhu L, Xie Y, Li F, Xiao X, Ma Z, et al. Bacillus licheniformis SA03 Confers Increased Saline-Alkaline Tolerance in Chrysanthemum Plants by Induction of Abscisic Acid Accumulation. Front Plant Sci. 2017;8:1143. Epub 2017/07/15. doi: 10.3389/fpls.2017.01143 ; PubMed Central PMCID: PMC5489591. 28706529 PMC5489591

[42] Liu J, Tang L, Gao H, Zhang M, Guo C. Enhancement of alfalfa yield and quality by plant growth-promoting rhizobacteria under saline-alkali conditions. J Sci Food Agric. 2019;99(1):281–9. doi: 10.1002/jsfa.9185 29855046

[43] Li H, Qiu Y, Yao T, Ma Y, Zhang H, Yang X. Effects of PGPR microbial inoculants on the growth and soil properties of Avena sativa, Medicago sativa, and Cucumis sativus seedlings. Soil and Tillage Research. 2020;199. doi: 10.1016/j.still.2020.104577

[44] Kapadia C, Sayyed RZ, El Enshasy HA, Vaidya H, Sharma D, Patel N, et al. Halotolerant Microbial Consortia for Sustainable Mitigation of Salinity Stress, Growth Promotion, and Mineral Uptake in Tomato Plants and Soil Nutrient Enrichment. Sustainability. 2021;13(15). doi: 10.3390/su13158369

[45] Miao T. Study on improvement of greenhouse soil by combination of microbial inoculum and straw fertilizer [Master]: Jilin Agricultural University; 2017.

[46] Zhang J. Breeding of engineering bacteria and developing of speed rot agents using for straw degradation in northeast China [Doctor]: Jilin Agricultural University; 2012.

[47] Wang T, Chen Y, Zhang M, Chen J, Liu J, Han H, et al. Arabidopsis AMINO ACID PERMEASE1 Contributes to Salt Stress-Induced Proline Uptake from Exogenous Sources. Front Plant Sci. 2017;8:2182. doi: 10.3389/fpls.2017.02182 29312416 PMC5743684

[48] Khalofah A, Migdadi H, El-Harty E. Antioxidant Enzymatic Activities and Growth Response of Quinoa (Chenopodium quinoa Willd) to Exogenous Selenium Application. Plants (Basel). 2021;10(4). doi: 10.3390/plants10040719 33917228 PMC8068041

[49] Zhou W, Leul M. Uniconazole-induced tolerance of rape plants to heat stress in relation to changes in hormonal levels, enzyme activities and lipid peroxidation. Plant Growth Regulation. 1999;27(2):99–104.

[50] Wang S, Tian H, Liu J, Pan S. Pattern and change of soil organic carbon storage in China: 1960s–1980s. Tellus B. 2003;55(2):416–27. doi: 10.1034/j.1600-0889.2003.00039.x

[51] Dorich RA, Nelson DW. Evaluation of Manual Cadmium Reduction Methods for Determination of Nitrate in Potassium Chloride Extracts of Soils. Soil Science Society of America Journal. 1984;48:1(1):72–5.

[52] Olsen SR, Sommers LE. 1Phosphorus Methods of Soil Analysis Part. 2: Chemical and microbiological properties. 1982.

[53] Knudsen D, Peterson GA, Pratt PF. Lithium, Sodium, and Potassium: Methods of Soil Analysis.

[54] Oksanen J, Kindt R, Legendre P, O’Hara B, Simpson G, Solymos P, et al. The VEGAN Package: community ecology package. 2008.

[55] Wang H, Takano T, Liu S. Screening and Evaluation of Saline–Alkaline Tolerant Germplasm of Rice (Oryza sativa L.) in Soda Saline–Alkali Soil. Agronomy. 2018;8(10). doi: 10.3390/agronomy8100205

[56] Eynard A, Lal R, Wiebe K. Crop Response in Salt-Affected Soils. Journal of Sustainable Agriculture. 2005;27(1):5–50. doi: 10.1300/J064v27n01_03

[57] Shi Y-l Liu X-r, Gao P-l Zhang Q-w, Zhang A-p Yang Z-l. Effects of Biochar and Organic Fertilizer on Saline-alkali Soil N2O Emission in the North China Plain. Huanjing Kexue. 2017;38(12):5333–43. doi: 10.13227/j.hjkx.201705035 29964598

[58] Wu L, Wang Y, Zhang S, Wei W, Kuzyakov Y, Ding X. Fertilization effects on microbial community composition and aggregate formation in saline‐alkaline soil. Plant and Soil. 2021;463(1):523–35. doi: 10.1007/s11104-021-04909-w

[59] Kong L, Gong X, Zhang X, Zhang W, Sun J, Chen B. Effects of arbuscular mycorrhizal fungi on photosynthesis, ion balance of tomato plants under saline-alkali soil condition. Journal of Plant Nutrition. 2020;43(5):682–98. doi: 10.1080/01904167.2019.1701029

[60] Torbaghan ME, Lakzian A, Astaraei AR, Fotovat A, Besharati H. Salt and alkali stresses reduction in wheat by plant growth promoting haloalkaliphilic bacteria. Journal of Soil Science and Plant Nutrition. 2017;17(4):1058–73. doi: 10.4067/s0718-95162017000400016

[61] Suleman M, Yasmin S, Rasul M, Yahya M, Atta BM, Mirza MS. Phosphate solubilizing bacteria with glucose dehydrogenase gene for phosphorus uptake and beneficial effects on wheat. PLoS One. 2018;13(9):e0204408. doi: 10.1371/journal.pone.0204408 30240432 PMC6150522

[62] Ku Y, Xu G, Tian X, Xie H, Yang X, Cao C, et al. Root colonization and growth promotion of soybean, wheat and Chinese cabbage by Bacillus cereus YL6. PLoS One. 2018;13(11):e0200181. doi: 10.1371/journal.pone.0200181 30462642 PMC6248894

[63] Zheng BX, Ding K, Yang XR, Wadaan MAM, Hozzein WN, Penuelas J, et al. Straw biochar increases the abundance of inorganic phosphate solubilizing bacterial community for better rape (Brassica napus) growth and phosphate uptake. Sci Total Environ. 2019;647:1113–20. doi: 10.1016/j.scitotenv.2018.07.454 30180320

[64] An Y, Zhou P, Xiao Q, Shi D. Effects of foliar application of organic acids on alleviation of aluminum toxicity in alfalfa. Journal of Plant Nutrition and Soil Science. 2014;177(3):421–30. doi: 10.1002/jpln.201200445

[65] Ma Y, Guo L, Wang H, Bai B, Shi D. Accumulation, distribution, and physiological contribution of oxalic acid and other solutes in an alkali-resistant forage plant, Kochia sieversiana, during adaptation to saline and alkaline conditions. Journal of Plant Nutrition and Soil Science. 2011;174(4):655–63. doi: 10.1002/jpln.201000337

[66] Wang X, Geng S, Ri Y-J, Cao D, Liu J, Shi D, et al. Physiological responses and adaptive strategies of tomato plants to salt and alkali stresses. Scientia Horticulturae. 2011;130(1):248–55. doi: 10.1016/j.scienta.2011.07.006

[67] Xiang G, Ma W, Gao S, Jin Z, Yue Q, Yao Y. Transcriptomic and phosphoproteomic profiling and metabolite analyses reveal the mechanism of NaHCO3-induced organic acid secretion in grapevine roots. BMC Plant Biology. 2019;19(1):383. doi: 10.1186/s12870-019-1990-9 31481025 PMC6724372

[68] Song J, Markewitz D, Wu S, Sang Y, Duan C, Cui X. Exogenous Oxalic Acid and Citric Acid Improve Lead (Pb) Tolerance of Larix olgensis A. Henry Seedlings. Forests. 2018;9(9). doi: 10.3390/f9090510

[69] Han Y, Wu X, Gu J, Zhao J, Huang S, Yuan H, et al. Effects of organic acids on the photosynthetic and antioxidant properties and accumulations of heavy metals of Melilotus officinalis grown in Cu tailing. Environ Sci Pollut Res Int. 2016;23(18):17901–9. doi: 10.1007/s11356-016-6920-x 27255310

[70] Chen HC, Zhang SL, Wu KJ, Li R, He XR, He DN, et al. The effects of exogenous organic acids on the growth, photosynthesis and cellular ultrastructure of Salix variegata Franch. Under Cd stress. Ecotoxicol Environ Saf. 2020;187:109790. doi: 10.1016/j.ecoenv.2019.109790 31639642

[71] Guo H, Chen H, Hong C, Jiang D, Zheng B. Exogenous malic acid alleviates cadmium toxicity in Miscanthus sacchariflorus through enhancing photosynthetic capacity and restraining ROS accumulation. Ecotoxicol Environ Saf. 2017;141:119–28. doi: 10.1016/j.ecoenv.2017.03.018 28324818

[72] Li S, Le S, Wang X, Bai J, Wang R, Zhao Y. Functional Analysis of Organic Acids on Different Oilseed Rape Species in Phytoremediation of Cadmium Pollution. Plants (Basel). 2020;9(7). doi: 10.3390/plants9070884 32668773 PMC7412029

